# p65BTK is a novel potential actionable target in KRAS-mutated/EGFR-wild type lung adenocarcinoma

**DOI:** 10.1186/s13046-019-1199-7

**Published:** 2019-06-14

**Authors:** Federica Giordano, Valentina Vaira, Diego Cortinovis, Sara Bonomo, Joyce Goedmakers, Federica Brena, Annamaria Cialdella, Leonarda Ianzano, Irene Forno, Maria Grazia Cerrito, Roberto Giovannoni, Gian Luca Ferri, Ennio Tasciotti, Silve Vicent, Francesco Damarco, Silvano Bosari, Marialuisa Lavitrano, Emanuela Grassilli

**Affiliations:** 1 Department of Medicine and Surgery, University of Milano-Bicocca, Monza, Italy; 2 Division of Pathology, Fondazione IRCCS Ca’ Granda Ospedale Maggiore Policlinico, Milan, Italy; 3 Department of Pathophysiology and Transplantation, University of Milan, Milan, Italy; 4 Medical Oncology Unit, San Gerardo Hospital, Monza, Italy; 5 Department of Biomedical Science, NEF-Laboratory, University of Cagliari, Cagliari, Italy; 6 Center for Biomimetic Medicine, Houston Methodist Research Institute, Houston, TX USA; 7 Houston Methodist Orthopedic and Sports Medicine, Houston Methodist Hospital, Houston, TX USA; 8 Center for Applied Medical Research, Program in Solid Tumors and Biomarkers, University of Navarra, Pamplona, Spain; 9 Department of Pathology, Anatomy and Physiology, University of Navarra, Pamplona, Spain; 10 IdiSNA, Navarra Institute for Health Research, Pamplona, Spain; 11 Centro de Investigación Biomédica en Red de Cáncer (CIBERONC), Madrid, Spain; 12 Division of Thoracic Surgery and Lung Tranplantation, Fondazione IRCCS Ca’ Granda Ospedale maggiore Policlinico Milano, Milano, Italy; 13 Present address: Department of Biology, University of Pisa, Pisa, Italy

**Keywords:** NSCLC, Drug resistance, p65BTK, BTK inhibitors, EGFR, EGFR inhibitors, Targeted therapy, Chemotherapy

## Abstract

**Background:**

Lung cancer is still the main cause of cancer death worldwide despite the availability of targeted therapies and immune-checkpoint inhibitors combined with chemotherapy. Cancer cell heterogeneity and primary or acquired resistance mechanisms cause the elusive behaviour of this cancer and new biomarkers and active drugs are urgently needed to overcome these limitations. p65BTK, a novel isoform of the Bruton Tyrosine Kinase may represent a new actionable target in non-small cell lung cancer (NSCLC).

**Methods:**

p65BTK expression was evaluated by immunohistochemistry in 382 NSCLC patients with complete clinico-pathological records including smoking habit, ALK and EGFR status, and in metastatic lymph nodes of 30 NSCLC patients. NSCLC cell lines mutated for p53 and/or a component of the RAS/MAPK pathway and primary lung cancer-derived cells from *Kras/Trp53* null mice were used as a preclinical model. The effects of p65BTK inhibition by BTK Tyrosine Kinase Inhibitors (TKIs) (Ibrutinib, AVL-292, RN486) and first-generation EGFR-TKIs (Gefitinib, Erlotinib) on cell viability were evaluated by MTT. The effects of BTK-TKIs on cell growth and clonogenicity were assessed by crystal violet and colony assays, respectively. Cell toxicity assays were performed to study the effect of the combination of non-toxic concentrations of BTK-TKIs with EGFR-TKIs and standard-of-care (SOC) chemotherapy (Cisplatin, Gemcitabine, Pemetrexed).

**Results:**

p65BTK was significantly over-expressed in EGFR-wild type (wt) adenocarcinomas (AdC) from non-smoker patients and its expression was also preserved at the metastatic site. p65BTK was also over-expressed in cell lines mutated for KRAS or for a component of the RAS/MAPK pathway and in tumors from *Kras/Trp53* null mice. BTK-TKIs were more effective than EGFR-TKIs in decreasing cancer cell viability and significantly impaired cell proliferation and clonogenicity. Moreover, non-toxic doses of BTK-TKIs re-sensitized drug-resistant NSCLC cell lines to both target- and SOC therapy, independently from EGFR/KRAS status.

**Conclusions:**

p65BTK results as an emerging actionable target in non-smoking EGFR-wt AdC, also at advanced stages of disease. Notably, these patients are not eligible for EGFR-TKIs-based therapy due to a lack of EGFR mutation. The combination of BTK-TKIs with EGFR-TKIs is cytotoxic for EGFR-wt/KRAS-mutant/p53-null tumors and BTK-TKIs re-sensitizes drug-resistant NSCLC to SOC chemotherapy. Therefore, our data suggest that adding BTK-TKIs to SOC chemotherapy and EGFR-targeted therapy may open new avenues for clinical trials in currently untreatable NSCLC.

**Electronic supplementary material:**

The online version of this article (10.1186/s13046-019-1199-7) contains supplementary material, which is available to authorized users.

## Background

Lung cancer accounts for about 28% of all cancer-related deaths worldwide and represents the number one killer cancer [1]. Moreover, the majority of cases are diagnosed at advanced stages of disease, thus inadequate for surgery [2, 3]. The main histotype is non-small cell lung cancer (NSCLC; 85% of cases), which in turn comprehends adenocarcinomas (AdC), squamous cell (SCC) and large cell (LCC) carcinomas [4].

In the evolution toward a more personalized and efficient therapeutic approach, particular emphasis is on the understanding of lung cancer biology and on the consequent identification of new actionable targets in order to develop a tailored medicine. Indeed, significant improvements have been achieved in molecular characterization of NSCLC, in particular of AdC. The most frequent driver mutations occur in receptors or protein kinases related to RAS/MAPK, PI3K/AKT/mTOR and JAK/STAT pathways, all of which eventually result in hyper-activation of the MAPK signaling [5, 6]. In particular, the most commonly mutated genes are, p53, KRAS, epidermal growth factor receptor (EGFR), mesenchymal epithelial transition factor (MET) and anaplastic lymphoma kinase (ALK) [7]. The definition of the mutational landscape of NSCLC has allowed the identification of actionable cancer genes such as EGFR, ALK, ROS1, BRAF and the development of targeted therapies [7]. Nevertheless, the percentage of patients without alterations in actionable genes is > 40% [8], making those patients inadequate for targeted therapy. Moreover, despite mutated KRAS is one of the most frequent (31%) alteration in NSCLC, it represents an unmet clinical need since no specific inhibitor has successfully progressed through clinical trials so far [9]. Notably, mutations in KRAS are mutually exclusive with EGFR mutations and are associated with severe prognosis and resistance to chemotherapy or EGFR inhibitors [10–12]. Finally, tumour primary or acquired resistance to target therapy regimens and even to chemotherapy severely impacts on NSCLC progression and patients’ prognosis [6, 13]. In this scenario, alternative molecular targets downstream of KRAS are urgently needed.

Bruton tyrosine kinase (BTK) is a 77 kDa non-receptor tyrosine kinase that plays a crucial role in B-cell activation, proliferation, maturation, differentiation and survival [14]. BTK has emerged as a novel molecular target in some B-cell leukemias and lymphomas where it is commonly overexpressed [15]. Accordingly, Ibrutinib, the first irreversible BTK inhibitor, has been recently approved by the FDA for the treatment of certain B-cell malignancies [16]. This has led to a rapid development in the field and several other BTK inhibitors, among which AVL-292 (Spebrutinib), are currently in advanced phase of clinical trial for different types of leukemia [17].

Recently, our lab identified and characterized p65BTK, a novel isoform of BTK, overexpressed in colon cancers. Notably, in this tissue only the messenger encoding p65BTK and not that for p77 is expressed [18]. Interestingly, p65BTK expression and abundance are post- transcriptionally regulated by the MAPK pathway and the protein acts downstream of KRAS. In addition, p65BTK is an obligate effector of RAS-mediated transformation [18], making it an attractive therapeutic target for KRAS-mutated cancers. To define a novel possible druggable target in cases currently not treatable with available targeted therapies, we investigated p65BTK expression in NSCLC studying the biological effects of its inhibition alone or in combination with SOC and targeted therapies in preclinical models of KRAS-mutated drug-resistant NSCLCs.

## Materials and methods

### Lung Cancer patients

A previously described series of 383 chemo- and/or radio-naïve NSCLC patients who underwent surgery for therapeutic purposes at Fondazione IRCCS Ca′ Granda-Ospedale Maggiore Policlinico Hospital (Milan, Italy) between 2004 and 2010 [19] was used to investigate p65BTK expression and correlation with patients’ clinic-pathological features. In addition, metastatic lymph nodes from 30 NSCLC patients were retrieved and analysed.

Patients’ informed consent was obtained and the study was approved by the Fondazione IRCCS Ca′ Granda Institutional Review Board (Institutional Review Board 179/2013). Data were analysed anonymously. Clinico-pathological records were available for the entire cohort whereas smoking habits was available for 348 patients. Moreover, the presence of ALK rearrangements or EGFR mutations was analysed for all cases as described [19]. Patients’ features are described in Additional file 1: Table S1.

### Antibody production and characterization

BN30 polyclonal antibody was obtained in rabbits by immunization with the N-terminal decapeptide of p65BTK, conjugated to keyhole limpet hemocyanin via an additional C-terminal cysteine residue and validated as follows: specificity of BN30 polyclonal antiserum (IgG fraction), used for IHC, was assessed by western blot analysis on lysates of SW480 cells transfected with control (luc) or p65BTK-specific siRNA and by immunocytochemistry, on sections from cell blocks of SW480 p65BTK-expressing and p65BTK-silenced cells (Additional file 1: Figure S1a, b). Moreover, its specificity was assessed also by western blot analysis on lysates of SW480 cells transfected with control (luc) or p65BTK-specific siRNA vs lysates from the lymphoblastic leukemia cell line Nalm-6 which express p77BTK together with low levels of p65BTK (Additional file 1: Figure S1c) [18].

### Tissue microarray (TMA) construction and immunohistochemical (IHC) staining

Representative tissue blocks of tumour and non-neoplastic lung tissue derived from each patient were used to construct TMAs, as previously described [20]. Briefly, for all lung cancer samples five representative cores were selected by a pathologist whereas for non-neoplastic parenchyma one core was chosen. Metastatic lymph nodes of NSCLC patients were analysed as well (*n* = 30) as full sections. For all blocks, 4-μm-thick sections were cut and subjected to IHC staining for p65BTK using the above described BN30 antibody in a BenchMark Ultra automatic system (Ventana Medical Systems). Reactions were revealed using the UltraView Universal DAB, according to the manufacturer’s instructions (Ventana Medical Systems) and all slides were counterstained with hematoxylin. As positive control we used a colon carcinoma specimen, whereas negative controls were prepared in the absence of primary antibody and included in each reaction. p65BTK cytoplasmic staining was evaluated and scored in all cases, by two pathologists independently, as percentage of positive neoplastic cells in all tumour cores or in the whole section (for metastatic lymph nodes).

### Cell lines, culture, and treatments

All commercial human NSCLC cell lines used were from ATCC. The mutational background of the four cell lines used for the in vitro experiments is reported in Table 2. Mouse lung cancer primary cell lines were from Silve Vicents’ lab [21]. Upon reception, cells were expanded and frozen as seed stocks of first or second passage. All cells were passaged for a maximum of 3 or 4 weeks, after which new seed stocks were thawed for experimental use. All cells were grown at 37 °C in 5% CO_2_ and were maintained as a sub confluent monolayer using the following media: Dulbecco’s modified eagle’s medium (DMEM) for SK-LU-1 and Calu-6 supplemented with non-essential amino acids (NEAA) and 1% sodium pyruvate; Roswell Park Memorial Institute 1640 (RPMI 1640) supplemented with 1% sodium pyruvate for NCI-H1975 and NCI-H2228. Mouse lung cancer primary cell lines were grown in DMEM. In addition, all media were also supplemented with 10% fetal bovine serum and 1% penicillin-streptomycin. Media, serum, and supplements were all from Invitrogen. BTK inhibitors Ibrutinib, AVL-292, RN486 and EGFR inhibitors Erlotinib and Gefitinib and MEK inhibitor Trametinib (all inhibitors were purchased from Selleckchem), were dissolved in DMSO and stored in aliquots at − 80 °C. Chemotherapeutic drugs Cisplatin, Pemetrexed and Gemcitabine were kindly provided by S. Gerardo Hospital (Monza). Caspase inhibitor QVD-OPh was Sigma-Aldrich.

### Western blot analysis

Protein extracts were prepared using high-salt lysis buffer (50 mM Hepes (pH 7.5), 500 mM NaCl, 1 mM DTT, 1 mM EDTA, 0.1% NP-40) supplemented with 1% protease inhibitor cocktail (Sigma-Aldrich). 20 μg of cell lysates were separated on 10% tris-glycine Wedge-wells gels (Invitrogen), transferred onto a nitrocellulose membrane (Invitrogen) and incubated with the following antibodies: anti-p65BTK BN49 [18]; anti-Actin (#A1978, Sigma-Aldrich); anti-vinculin (#V9131, Sigma-Aldrich); anti-pERK (#4370, Cell Signaling Technology); BTK(#611117, Becton Dickinson). Purified p77BTK (#B4312) was from Sigma-Aldrich. Images were acquired using G:BOX XT4 Chemiluminescence and Fluorescence Imaging System (Syngene) and processed with Adobe Photoshop.

### Cell proliferation/viability assay

Cells were seeded in 96-well plate at 70% confluency in octuplicates for overnight attachment. For survival curves, cells were treated with the different concentrations of inhibitors (day 0) and cell number was evaluated after 72 h using an MTT-based assay (Sigma-Aldrich) according to the manufacturer’s instructions. For growth curves, 3000 cells were seeded and their number was evaluated at 0, 24, 48 and 72 h by crystal violet staining. Briefly, after washing with PBS, cells were fixed with Formalin 10% (Bio-Optica) for 1 h on the shaker and then stained with a crystal violet solution (Sigma-Aldrich) in 35% ethanol (Sigma-Aldrich) for 20 min at room temperature. After washing extensively with tap water, color was extracted by adding 0.1 M acetic acid and quantified by spectrophotometer at 595 nm. Graphs represent the average of 3 to 5 independent experiments. Average ± s.e.m. are plotted in the graphs.

### Cell toxicity assay

Cells were seeded in octuplicates at 70% confluency and the next morning treated or not with drugs and inhibitors and their combination as indicated in the figures. Cell viability was evaluated by CellTiter-Glo® Luminescent Cell Viability Assay (Promega) following manufacturer’s instructions. Graphs represent the average of 3 to 5 independent experiments. Average ± s.e.m. are plotted in the graphs.

### Analysis of synergy

Combination Index (CI) was calculated as reported by Fransson et al. [22]. Predicted cell viability (PCV) (%) was calculated according to the following formula: PCV (%) = cell viability after treatment with drug 1 (%) x cell viability after treatment with drug 2 (%) × 0.01. CI was then derived as the ratio of the measured cell viability of the cells incubated with both drugs /PCV. 0.8 < CI < 1.2 = additive effect (the interval of 1.0 ± 0.2 is set to account for intra-assay variability); CI < 0.8 = synergistic effect; CI > 1.2 = sub-additive effect. If the measured cell viability for a combination of two drugs is higher than the cell viability for one or both of the drugs, the effect is considered antagonistic. CI < 0.5 has been considered as a strong synergistic effect.

### Caspase assay

2 × 10^4^ cells/well were seeded in triplicate in 96-well plates, let adhere overnight, and treated for 24hs before evaluating active caspase-3/7 by Caspase-Glo3/7 Assay System (Promega, Milan, Italy) according to the manufacturer’s instructions. Assays were repeated 3 times for each time point (*n* = 3).

### Colony forming assay

Cells were seeded at low density (1000 cells/well in 6-well plate) in triplicate and left untreated or treated with different concentrations of Ibrutinib, AVL-292 and RN486. Medium (alone or supplemented with the inhibitors) was replaced every 3 days. After 10 days, colonies were fixed and stained with 1% crystal violet in 35% ethanol. Images were acquired using G:BOX XT4 Chemiluminescence and Fluorescence Imaging System (Syngene, Cambridge, UK) and processed with Adobe Photoshop. Colony assays were repeated 3 times.

### Immunofluorescence staining

NSCLC cell lines were seeded at a density of 10× 10^5^ cells/well on glass slides pretreated with Polylysine (Sigma) and grown for 2 days. After treatments cells were washed with PBS and fixed for 10 min in 1.6% Paraformaldehyde (Sigma-Aldrich) and washed again with PBS. The slides were then permeabilized with ice-methanol at − 80 °C overnight. The day after, slides were incubated with anti-pBTK tyr551 (1:100 in 3% BSA in PBS; Bioss) for 1 h at room temperature and then washed 3 times with PBS. Then, the slides were incubated with secondary antibodies diluted 1:2000 in 3% BSA in PBS for 40 min at room temperature, washed 3 times with PBS and evaluated using a fluorescence microscope (Zeiss, Germany). Nuclei were counterstained with DAPI (Sigma-Aldrich).

### Statistical analysis

Data were analyzed using unpaired *t* test with or without Welch correction unless otherwise specified. A probability (p) value less than 0.05 was considered as statistically significant.

## Results

### p65BTK is overexpressed in advanced lung adenocarcinomas with wild type EGFR from never-smoker patients

**Fig. 1:**
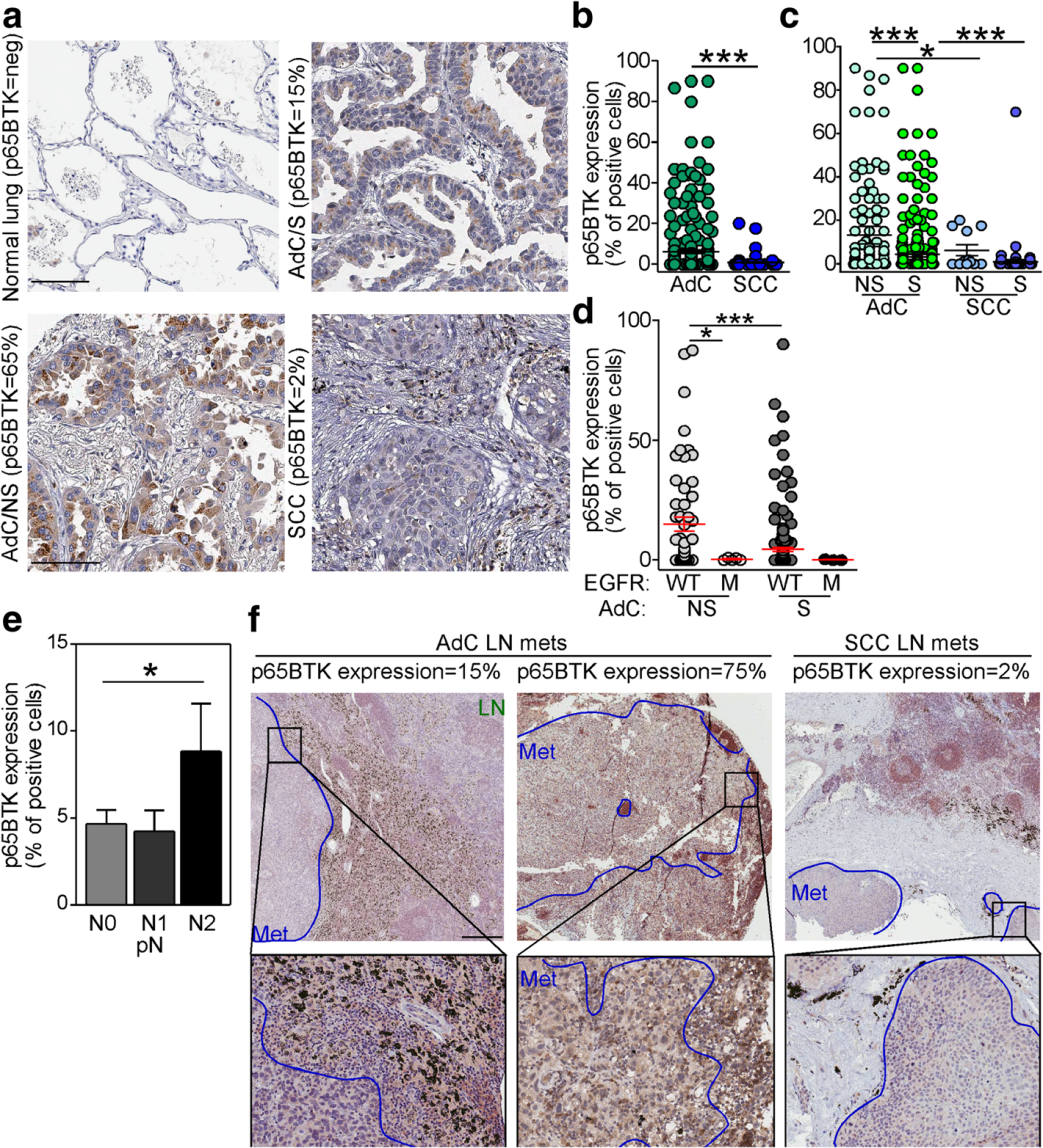
p65BTK is overexpressed in advanced lung adenocarcinomas with wild type EGFR from never-smoker patients. **a** IHC analysis of p65BTK in lung cancer tissue samples from a cohort of NSCLC patients using the BN30 antibody. Representative images of normal lung and lung cancer tissues are shown. SCC: squamous cell carcinoma; AdC/S: adenocarcinoma from smoker patient; AdC/NS: adenocarcinoma from non-smoker patient. Scale bar 100 μM. **b** Quantification of p65BTK expression in SCC and AdC patients. ***, *p* < 0.0001 by unpaired *t* test with Welch’s correction. **c** Quantification of p65BTK expression in smoker and non-smoker patients AdC and SCC patients. NS: non-smoker; S: smoker. Quantification of p65BTK expression**. d** Quantification of p65BTK expression in smoker and non**-**smoker AdC patients with either wild type (WT) or mutated (MT) EGFR. *, *p* = 0.04; ***, *p* < 0.0001 by non-parametric *t* test. **e** Quantification of p65BTK expression in primary NSCLC according to pN status. *, *p* = 0.04 by unpaired *t* test with Welch’s correction. **f** IHC analysis of p65BTK in metastatic lymph nodes of lung adenocarcinomas (AdC) or squamous cell carcinoma (SCC). Representative images show different expression levels of the kinase in the metastatic setting. Scale bars 500 μm (top panels) or 200 μm (lower panels)

Using the BN30 isoform-specific polyclonal antibody we previously developed and characterized in the lab we examined p65BTK expression in cancer tissues derived from a cohort of chemo- and/or radio-naïve NSCLC patients (Additional file 2: Table S1). To this end, 382 out of 383 cases were available. Overall, p65BTK was expressed in 51% of NSCLC (Table 1). Interestingly, p65BTK was more expressed in AdC than in SCC cases (*p* < 0.0001; Fig. 1a and b, Table 1). Within AdC patients, the protein’s levels were significantly higher in never-smokers and in EGFR-wt tumors (p < 0.0001; Fig. 1c and d, Table 1). Conversely, no difference was observed according to ALK translocation (Additional file 3: Figure S2). When we analyzed p65BTK expression according to nodal status of NSCLC patients, we found that tumor from patients with distant nodal metastases (e.g., pN2) expressed higher levels of the protein than tumors with loco-regional or no nodal involvement (pN1 and pN0, respectively) (Fig. 1e). Finally, p65BTK expression was present also in nodal metastases at levels comparable to the primary tumour (Fig. 1f). Interestingly, p65BTK staining was observed in in both cancer cells and tumor-infiltrating lymphocytes (TILs) in the lymph nodes metastasis. It is known that than 80% of TILs is represented by T cells [23] and that p77BTK is not expressed in the T cell lineage [14]. We showed that p65BTK is expressed in T cells lysates from FACS-purified T cells using BN49 antibody (Additional file 4: Figure S3), thus suggesting that in the nodal metastases the reactivity was due to expression of p65BTK. Overall, these data suggest that p65BTK could be a novel target in advanced NSCLC from EGFR-wt non-smokers that are not eligible for targeted therapy.

**Table 1:** p65BTK score of the NSCLC patients’ tissue analyzed by IHC

p65BTK expression	All (382)	AdC (293)	SCC (89)	Smokers (283)	Non-smokers (65)
Negative	**189 (49.3%)**	**129 (43.9%)**	**60 (67.4%)**	**151 (53.3%)**	**18 (27.7%)**
1–10% positive cells	131 (34.2%)	106 (36%)	25 (28.1%)	101 (35.7%)	21 (32.3%)
11–20% positive cells	12 (3.1%)	10 (3.4%)	2 (2.2%)	5 (1.8%)	5 (7.7%)
21–50% positive cells	24 (6.3%)	23 (7.8%)	1 (1.1%)	15 (5.3%)	8 (12.1%)
51–100% positive cells	26 (6.8%)	25 (8.5%)	1 (1.1%)	11 (3.9%)	13 (20%)
Positive	**193(50.4%)**	**164 (55.8%)**	**29 (32.6%)**	**132 (46.7%)**	**47 (72.3%)**

The analysis was performed on TMA using the antibody BN30, produced and characterized in the lab. *AdC* adenocarcinoma, *SCC* squamous cell carcinoma

In bold are indicated the number of samples completely negative or positive (any positivity) for p65BTK expression

### NSCLC cells with activated KRAS express high levels of p65BTK

**Fig. 2:**
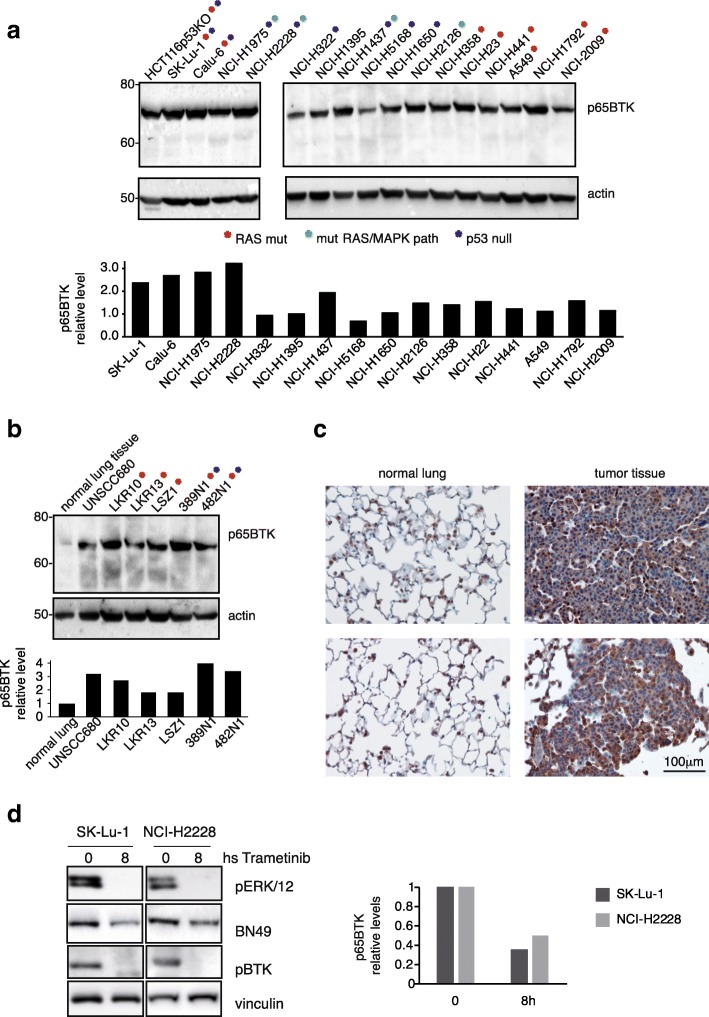
NSCLC cells with activated KRAS express high levels of p65BTK. **a** *Top:* Western Blot analysis of p65BTK expression in NSCLC human cell lines with different mutations along the RAS/MAPK pathway and in p53. Lysate from HCT116p53KO colon cancer cells was loaded as a positive control. *Bottom*: fold change of p65BTK protein expression in NSCLC cell lines normalized to beta actin, setting as expression level = 1 NCI-H1935 which do not possess mutations in KRAS or in the RAS/MAP pathway not in the p53 gene. **b** *Top:* Western Blot analysis of p65BTK expression in primary lung cancer cells derived from *KrasLSL-G12D* (LKR10, LKR13, LSZ1) and *K*r*asLSL-G12D;Trp53*^*f/f*^ (389 N1, 482 N1) mice. UNSCC680 is a primary cell line from a mouse squamous cell carcinoma. *Bottom*: fold change of p65BTK protein expression normalized to beta actin. In **a** and **b** p65BTK was detected by BN49 antibody [18] and beta actin was used as a loading control. **c** IHC analysis of p65BTK in normal and tumoral lung tissue samples from 2 different KrasLSLG12D; Trp53 ^f/f^ mice using BN30 antibody. **d** *Left*: Western Blot analysis of p65BTK expression in SK-Lu-1 and NCI-H2228 cells after treatment with the MEK inhibitor Trametinib (1 μM). *Right*: fold change of p65BTK protein expression normalized to vinculin

We then analysed p65BTK expression in NSCLC cell lines. By using the BN49 isoform-specific polyclonal antibody that we previously developed and characterized [18], we showed that p65BTK was abundantly expressed at the protein level by several NSCLC cell lines with a mutation in KRAS or in the RAS/MAPK pathway (Fig. 2a). In particular, the highest levels of p65BTK were expressed by cell lines with both a p53 mutation and a mutation in KRAS or in the RAS/MAPK pathway. The highest expressing cell lines, ie KRAS-mutated Calu-6 and SK-Lu-1, EGFR-doubly mutated NIH-H1975, and ALK-translocated NIH-H2228 were analysed by qPCR for p65BTK and p77BTK expression. Interestingly, only p65BTK-encoding transcript was expressed by all cell lines (Additional file 5: Table S2), confirming our previous data from colorectal carcinoma [18].

p65BTK was overexpressed in primary cell lines (LKR10 and LKR13) (Fig. 2b) derived from tumors spontaneously arising in *KrasLSL-G12D* mice, a genetically engineered mouse model of *Kras*-driven lung cancer [24]. Notably, its expression was increased in primary cell lines (389 N1 and 482 N1) derived from tumors of *KrasLSL-G12D;Trp53*^*f/f*^ mice (Fig. 2b), where p53 inactivation accelerates AdC progression in a *Kras*-mutated context [24]. Remarkably, compared with non-neoplastic tissue, p65BTK was uniformly and highly expressed in tumor samples from *Kras LSL-G12D;Trp53*^f/f^ mice (Fig. 2c).

Finally, accordingly to what we previously reported for colon cancer cells [18] we confirmed that p65BTK expression is regulated by the activation of the RAS/MAPK pathway by showing that its expression, as well as its activation, is down-regulated upon the treatment of NSCLC cells with Trametinib, a MEK inhibitor (Fig. 2d).

These results confirm what observed in NSCLC patients and our previous data on colorectal cancer [18], regarding the preferential up-regulation of p65BTK in a context of RAS/MAPK hyper-activation. Moreover, these data show that high p65BTK expression correlates with advanced lung cancer.

### p65BTK targeting affects cell viability of NSCLC cell lines and tumor-derived primary cells scarcely responsive to EGFR inhibition

**Fig. 3:**
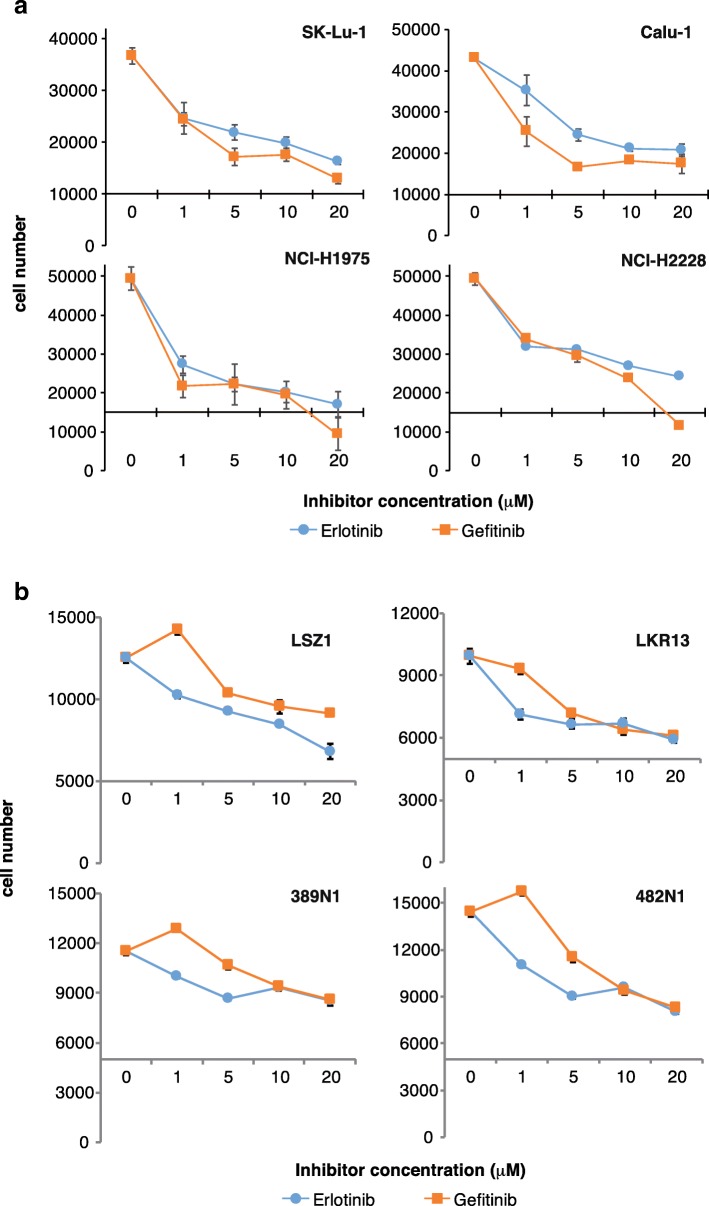
EGFR inhibition does not affect cell viability of NSCLC cell lines and tumor-derived primary cells with mutations along the EGFR/RAS/MAPK pathway. Dose-response curves of **a** human NSCLC cell lines (SK-Lu1, Calu-6, NCI-H1975 and NCI-H2228) and **b** primary lung cancer cell lines derived from *KrasLSL-G12D* (LSZ1, LKR13) and *KrasLSL-G12D;Trp53*^*f/f*^ mice, (389 N1, 482 N1) treated with increasing concentrations of EGFR inhibitors (Erlotinib and Gefitinib). Cell viability was evaluated by MTT assay. X-axis crosses in correspondence of T0 values (before starting the treatment); 72 h values are then expressed as the variation relative to the initial cell number. Scale on Y-axis is adapted to the different growth rates shown by each cell line. Data are presented as mean ± SEM. *n* ≥ 3 independent experiments

**Fig. 4:**
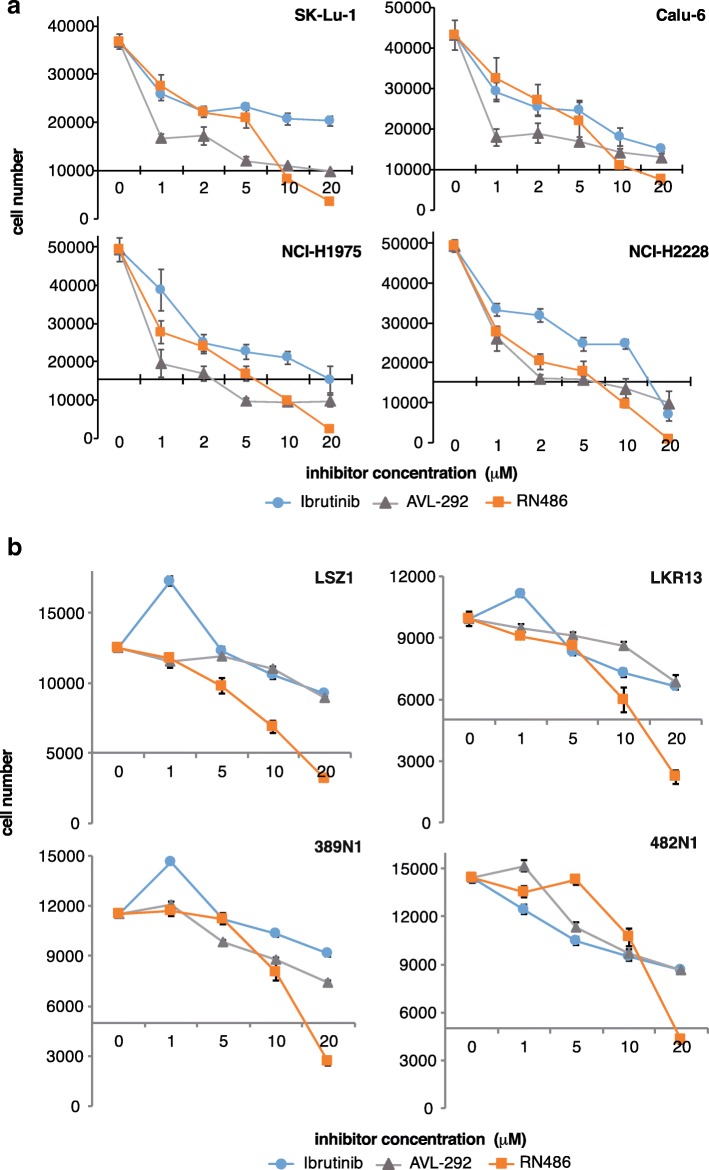
p65BTK targeting affects cell viability of NSCLC cell lines and tumor-derived primary cells scarcely responsive to EGFR inhibition. Dose-response curves of **a** human NSCLC cell lines (SK-Lu1, Calu-6, NCI-H1975 and NCI-H2228) and **b** primary lung cancer cells derived from *KrasLSL-G12D* (LSZ1, LKR13) and *KrasLSL-G12D;Trp53*^*f/f*^ mice (389 N1, 482 N1) treated with increasing concentrations of BTK inhibitors (Ibrutinib, AVL-292, RN486). Cell viability was evaluated by crystal violet staining. X-axis crosses in correspondence of T0 values (before starting the treatment); 72 h values are then expressed as the variation relative to the initial cell number. Scale on Y-axis is adapted to the different growth rates shown by each cell line. Data are presented as mean ± SEM. *n* ≥ 3 independent experiments

To investigate whether p65BTK could be a novel target in advanced NSCLC, we firstly evaluated whether p65BTK was constitutively activated in NSCLC cell lines with different mutations along the EGFR/RAS/MAPK pathway (Table 2). To this end, we checked the activation of p65BTK analysing its phosphorylation in Y465. This tyrosine residue corresponds to Y551 in p77BTK, whose phosphorylation reflects its activation [25]. Our results indicated that in all the cell lines expressing high levels of p65BTK the kinase was constitutively active (Additional file 6: Figure S4, panel a, b) and its activation was dampened by BTK inhibitors (Additional file 7: Figure S4, panel c, d). We also tested the effects of EGFR inhibition in the same cell lines by treating them with increasing concentrations of the two specific first generation EGFR-TKIs currently used in therapy, Erlotinib and Gefitinib (Fig. 3a). After 72 h, only a dose-dependent reduction of cell number but no cytotoxic effects were observed. The same results were confirmed in tumor-derived primary cell lines from *KrasLSL-G12D* (LKR10 and LKR13) and *KrasLSL-G12D;Trp53*^*f/f*^ (389 N1 and 482 N1) mice (Fig. 3b). Next, we tested the effect of BTK inhibitors (Ibrutinib, AVL-292 and RN486) in the same cell lines and we found that p65BTK inhibition resulted in a stronger anti-proliferative effect than the treatment with EGFR inhibitors. In fact, a significant reduction in cell number was observed even at lower doses of BTK inhibitors, among which RN486 showed even cytotoxic effects in all cell lines when used at the highest concentration (Fig. 4a and b).

**Table 2:** Known genetic alterations characterizing the NSCLC lines used in the paper. Information about genetic defects were retrieved from the database of the Wellcome Trust Sanger Institute Catalogue Of Somatic Mutations In Cancer COSMIC, https://cancer.sanger.ac.uk/cell_lines)

Cell line	Hystotype	Mutational status			
		TP53	EGFR	KRAS	Other mutations/molecular alterations
Calu-6	anaplastic carcinoma	mut	wt	Q61K	JAK1, BRCA1, p16INK4A methylation
SK-Lu-1	adenocarcinoma	mut	wt	G12D	KIT, CSF3R, FLT4
NCI-H1975	adenocarcinoma	mut	L858R + T790 M	wt	PDGFRA, PIK3CA
NCI-H2228	adenocarcinoma	mut	wt	wt	ALK translocation, PDGFRA, RB

These results indicate that cell lines bearing mutations in the EGFR/RAS/MAPK pathway are very sensitive to p65BTK inhibition.

### p65BTK inhibition strongly impairs proliferation and clonogenicity of NSCLC cell lines

**Fig. 5:**
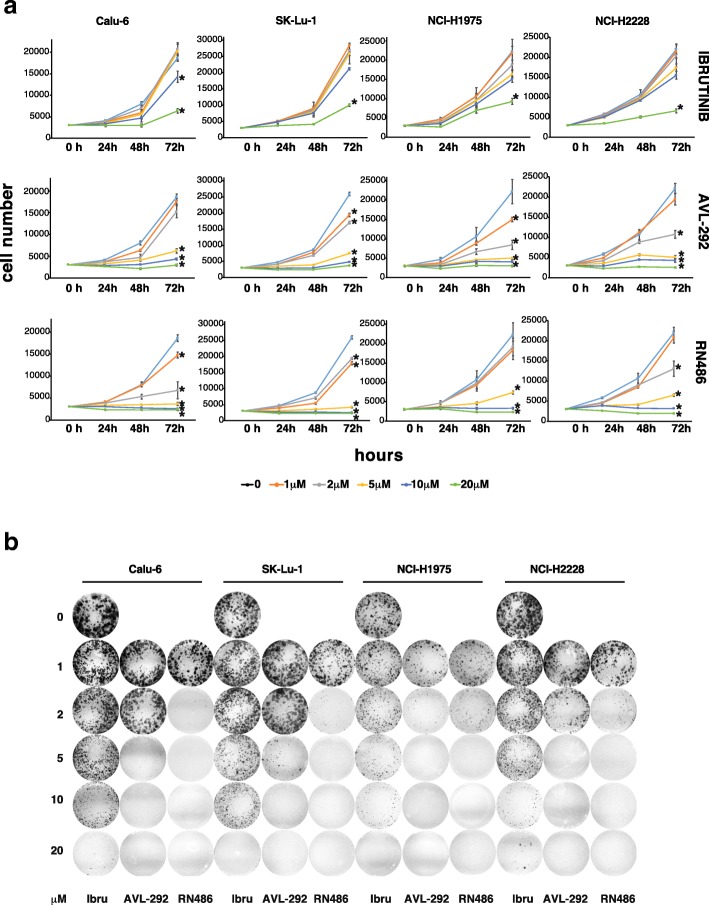
p65BTK inhibition strongly impairs proliferation and clonogenicity of NSCLC cell lines. **a** Growth curves of human p53-null NSCLC cell lines treated with increasing concentrations of BTK inhibitors; cell number was evaluated each 24 h by MTT assay. Scale on Y-axis is adapted to the different growth rates shown by each cell line. Data are presented as mean ± SEM. n ≥ 3 independent experiments**.** * indicates *p* < 0.05 vs untreated**. b** Clonogenicity assay of human p53-null NSCLC cell lines treated with increasing concentrations of BTK inhibitors for 10 days. A representative image of one experiment out of 3 is shown

Given the significant reduction in cell number obtained with BTK inhibitors, we then investigated the effects of p65BTK inhibition on cell proliferation and clonogenicity of NSCLC cell lines. First, we performed growth curves of NSCLC cell lines in the presence of increasing concentrations of p65BTK inhibitors and observed that p65BTK inhibition strongly impaired proliferation of all NSCLC cell lines. As shown in Fig. 5a, in all the cell lines analysed, 10 μM Ibrutinib caused a slight to moderate decrease of proliferation whereas a strong decrease was observed only at the highest dose of Ibrutinib (20 μM). AVL-292 and RN486 instead strongly decreased cell proliferation when used at concentrations as low as 5 μM (Fig. 5a).

Moreover, we investigated whether p65BTK inhibition could affect the clonogenicity of NSCLC cell lines by evaluating colonies growth in the presence of increasing concentrations of p65BTK inhibitors. Our results indicated that Ibrutinib was able to reduce colony formation only at the highest dose (20 μM), whereas AVL-292 and RN486 were efficient already at lower concentration (5 μM) (Fig. 5b). In particular, NCI-H1975 and NCI-H2228 cell lines, bearing a double mutation in the EGFR and an ALK translocation respectively, resulted more sensitive to all the inhibitors tested (Fig. 5b).

All together these data show that p65BTK inhibition impairs proliferation and clonogenicity of NSCLC cell lines, being AVL-292 and RN486 effective at low concentration.

### p65BTK inhibition sensitizes NSCLC cell lines scarcely responsive to target therapy and chemotherapy independently of the EGFR and KRAS status

**Fig. 6:**
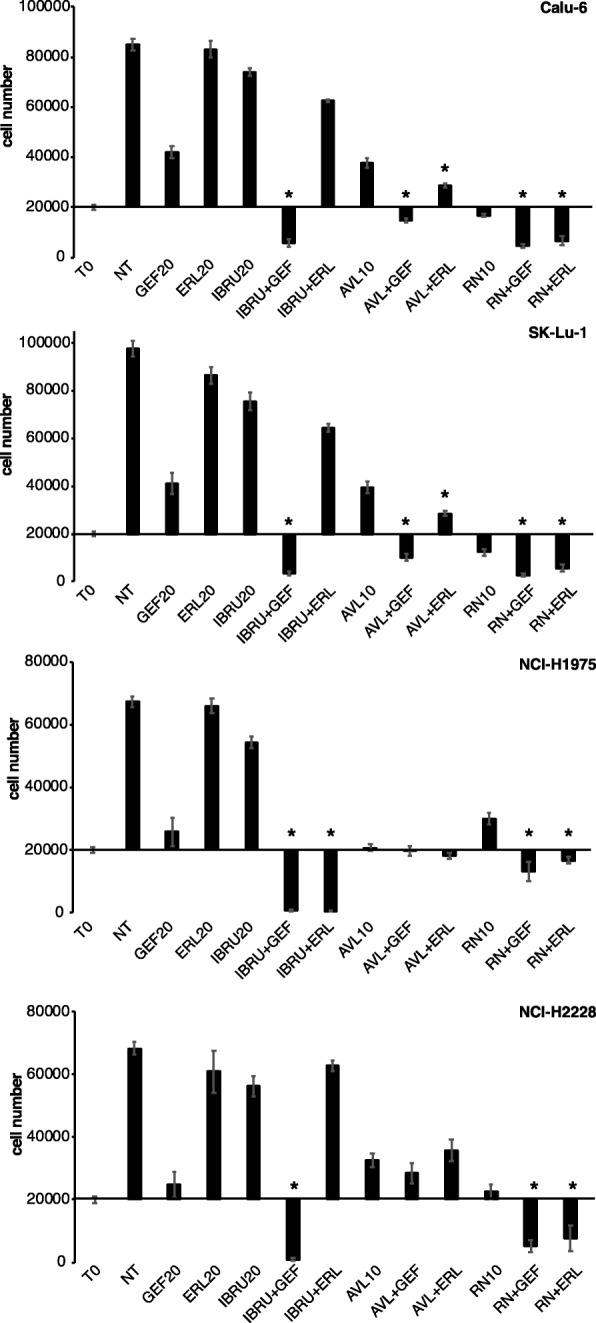
p65BTK inhibition sensitizes NSCLC cell lines scarcely responsive to EGFR-targeted therapy. Cell viability of human p53-null NSCLC cell lines in response to different combinations of BTK and EGFR inhibitors (T0 = time 0; NT = untreated; GEF20 = Gefitinib 20 μM; ERL20 = Erlotinib 20 μM. IBRU20 = Ibrutinib 20 μM; AVL10 = AVL-292 10 μM; RN10 = RN486 10 μM). X-axis crosses in correspondence of T0 values (before starting the treatment); 72 h values are then expressed as the percentage variation relative to the initial cell number. Scale on Y-axis is adapted to the different growth rates shown by each cell line. Data are presented as mean ± SEM. n ≥ 3 independent experiments. * indicates *p* < 0.05 vs T0 values

Next, we tested whether the addition of Ibrutinib, AVL-292 and RN486 could sensitize cells to EGFR inhibitors (Fig. 6). We showed that the combination of EGFR inhibitors and BTK inhibitors had a strong synergistic effect in most cases (Table 3). In particular, the combination of 20 μM Ibrutinib and 20 μM Gefitinib was highly cytotoxic for all the NSCLC cell lines scarcely responsive to EGFR inhibition. Whereas, the combination of 20 μM Ibrutinib with 20 μM Erlotinib was cytotoxic only for NCI-H1975 cells (which bear L858R + T790 M EGFR mutations). The combination of 10 μM AVL-292 with 20 μM Gefitinib was cytotoxic in SK-Lu-1 and Calu-6 cells, cytostatic in NCI-H1975 cells and ineffective in NCI-H2228 cell. On the contrary, the combination of 10 μM AVL-292 and 20 μM Erlotinib was mildly cytotoxic only in the NCI-H1975 cells. 10 μM RN486 in combination either with 20 μM Gefitinib or 20 μM Erlotinib was cytotoxic in all the cell lines.

**Fig. 7:**
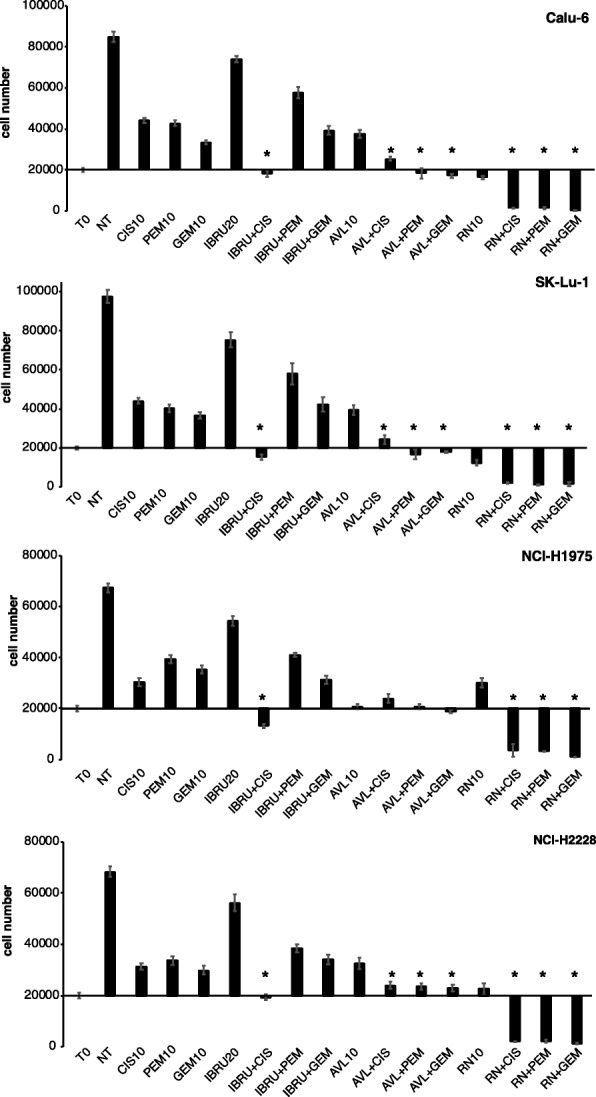
p65BTK inhibition reverts resistance of NSCLC cell lines to chemotherapy. Cell viability of human p53-null NSCLC cell lines in response to different combinations of BTK and SOC chemotherapeutic agents (T0 = time 0; NT = untreated; CIS10 = Cisplatin 10 μM; PEM10 = Pemetrexed 10 μM; GEM10 = Gemcitabine 10 μM; IBRU20 = Ibrutinib 20 μM; AVL10 = AVL-292 10 μM; RN10 = RN486 10 μM). X-axis crosses in correspondence of T0 values (before starting the treatment); 72 h values are then expressed as the percentage variation relative to the initial cell number. Scale on Y-axis is adapted to the different growth rates shown by each cell line. Data are presented as mean ± SEM. n ≥ 3 independent experiments. * indicates *p* < 0.05 vs T0 values

We then studied the effect of combining p65BTK inhibitors with SOC chemotherapy agents currently used in the clinic such Cisplatin, Pemetrexed and Gemcitabine, also in this case the combination of the different BTK inhibitors and chemotherapeutic drugs was mostly strongly synergistic (Table 4). In fact, the combination of RN486 with any of these drugs dramatically reverted the chemo-resistance in all the cell lines analysed. The co-treatment Ibrutinib/Cisplatin was cytotoxic only in SK-Lu-1 and NCI-H1975 cells whereas it had a cytostatic effect in Calu-6 and NCI-H2228 cells. The combination of Ibrutinib with Pemetrexed or Gemcitabine was ineffective. Finally, the combination of AVL-292 with SOC drugs had a stronger anti-proliferative effect than each of the drugs administered alone in all cell lines (Fig. 7).

**Table 3:** Synergism between BTK and EGFR inhibitors

	Ibrutinib		AVL-292		RN486	
	Gefitinib	Erlotinib	Gefitinib	Erlotinib	Gefitinib	Erlotinib
Calu-6	0.04	0.20	0.18	0.18	0.13	0.10
SK-Lu-1	0.02	0.20	0.13	0.17	0.11	0.11
NCI-H1975	0.01	0.01	0.74	0.26	0.34	0.17
NCI-H2228	0.01	ant	ant	ant	0.18	0.11

Combination Index (CI) was calculated as reported by Fransson (Fransson, A., et al. (2016) J Ovarian Res 9 [1]: 27) and detailed in material and methods

0.8 < CI < 1.2 = additive effect, CI < 0.8 = synergistic effect (CI < 0.5 = strong synergistic effect), CI > 1.2 = sub-additive effect, ant = antagonistic effect

**Table 4:** Synergism between BTK inhibitors and Chemoterapy

	Ibrutinib			AVL-292			RN486		
	CisPt	Pemetr	Gemcitab	CisPt	Pemetr	Gemcitab	CisPt	Pemetr	Gemcitab
Calu-6	0.11	ant	ant	0.3	0.23	0.28	0.04	0.05	0.01
SK-Lu-1	0.09	ant	ant	0.28	0.21	0.25	0.07	0.05	0.07
NCI-H1975	0.16	0.38	0.33	ant	0.51	0.52	0.08	0.05	0.02
NCI-H2228	0.22	0.41	0.41	0.47	0.43	0.47	0.06	0.06	0.03

Combination Index (CI) was calculated as reported by Fransson (Fransson, A., et al. (2016) J Ovarian Res 9 [1]: 27) and detailed in material and methods

0.8 < CI < 1.2 = additive effect, CI < 0.8 = synergistic effect (CI < 0.5 = strong synergistic effect), CI > 1.2 = sub-additive effect, ant = antagonistic effect

Finally, we determined that the cytotoxicity observed when combining p65BTK inhibition with EGFR inhibition or SOC therapy was due to induction of apoptosis as demonstrated by caspase activation and protection from cell death upon addition of the pan-caspase inhibitor QVD-OPh (Additional File 7: Figure S5).

Overall, the inhibition of p65BTK in combination with the EGFR-TKIs or chemotherapeutic drugs is effective in sensitizing NSCLC cells scarcely responsive to the current treatments, even if different inhibitors show or not synergy depending on which EGFR inhibitor or chemotherapeutic drug they are combined with.

## Discussion

In the last decade, significant advances at the molecular level have afforded an improved understanding of the underlying pathology and significant biological heterogeneity of NSCLC. Multiple signalling pathways have now been identified, as well as specific oncogenic driver mutations that lead to malignant transformations. Indeed, a number of clinical series has been profiled for the identification of key actionable alterations [6]. Despite the continuous discoveries in cancer treatment, the problem of the primary or acquired resistance is still unsolved. Therefore, it is of particular importance the identification of new molecular targets to overcome drug resistance of NSCLC.

Here we report that p65BTK is an emerging actionable target in NSCLC cells resistant to chemotherapy and scarcely responsive to target therapy. We previously demonstrated that BTK is an actionable target in KRAS-mutated colon cancer [18]. In this study we extended this observation to NSCLC and particularly to AdC and showed that p65BTK levels were significantly higher in EGFR-wt tumours from never-smoker patients and in tumour with metastasis at distant nodal stations (Fig. 1). Moreover, nodal metastases from NSCLC retained p65BTK expression, claiming a role for p65BTK also in advanced stage of disease. We confirmed that only p65BTK, and not p77BTK, was expressed in NSCLC (Additional file 5: Table S2) and we showed that p65BTK over-expression correlated with mutations in KRAS or the RAS/MAPK pathway both in in vitro and in vivo models of lung cancers with mutated KRAS (Fig. 2).

Importantly, BTK inhibition significantly hampered cell proliferation and clonogenicity in all the cells lines with hyper-activation of the MAPK pathway deriving from different genetic defects, such as a double mutation L858R/T790 M in the EGFR (NCI-H1975), an ALK translocation (NCI-H2228) or a mutation in KRAS (SK-Lu-1 and Calu-6) (Figs. 3, 4, 5). Moreover, BTK inhibition re-sensitized lung cancer cells to either EGFR-targeted (Fig. 6) or SOC chemo-therapies (Fig. 7) disregarding EGFR/KRAS mutational status.

Recently we demonstrated that p65BTK, a new oncogenic isoform of BTK different from the already known 77 kDa isoform, is highly expressed in colon cancer cells and tissues. We also demonstrated that BTK oncogenic activity is mediated only by the p65 isoform, and that p65BTK acts downstream of the RAS/MAPK pathway. In fact, p65BTK transforming activity depends on active signal-regulated protein kinases-1/2 (ERK1/2) and on RAS activity. Accordingly, p65BTK over-expression in colon cancer tissues correlates with ERK1/2 activation and its inhibition decreases cell growth and survival of colon cancer cells [18]. Here we confirm that, also in NSCLC, p65BTK expression level depends on the RAS/MAPK pathway activation (Fig. 2d) and that p65BTK inhibition strongly affect cell proliferation and survival (Figs. 4 and 5). Altogether, data from colorectal cancer and NSCLC suggest that p65BTK is an emerging actionable target in tumour cells resistant to chemotherapy and scarcely responsive to target therapy because of lack of EGFR mutation or presence of activated KRAS [26, 27]. Notably, despite KRAS mutation is one of the most prevalent oncogenic driver mutations in NSCLC (up to 31%), its targeting remains elusive, mainly because of the lack of molecules able to successfully pass the clinical trial step [28]. Indeed, there are no effective therapeutic approaches toward mutated KRAS and the possibility of targeting a downstream effector of KRAS, such as p65BTK, would therefore represent an alternative strategy for overcoming this main limitation.

BTK has been for long time considered to be exclusively expressed in hematopoietic cells, where it is crucial for B cell maturation and proliferation and for monocyte/macrophage activation [14]. BTK inhibitors were developed for the treatment of lymphoproliferative disorders: among them Ibrutinib is already FDA-approved for the treatment of mantle cell lymphoma, chronic lymphocytic leukemia and Waldenström macroglobulinemia. On the contrary, other BTK inhibitors, such as AVL-292, are in clinical trials for different hematological malignancies such as several B-cell leukemias/lymphomas, myelomas and acute myelogenous leukemia, and autoimmune diseases [29]. However, in recent years a number of reports demonstrated the expression of BTK in solid tumors and showed promising results by its inhibition with Ibrutinib [30–32]. Even though in many cases (such as glioma and glioblastoma, oesophageal and gastric cancers, renal cell and ovarian carcinoma) the isoform of the BTK expressed was not fully investigated and has been assumed to be p77, other reports pointed out the existence of different isoforms [32].

Due to their established effect on multiple tumour-related kinases such as the EGFR family members [33], Ibrutinib and AVL-292 have been experimentally proved useful in solid cancers such as glioblastoma, lung and breast carcinomas [34–39] and are now in clinical trials for EGFR-mutated NSCLC (https://clinicaltrials.gov/ct2/show/NCT02321540?term=ibrutinib&cond=NSCLC&rank=2) and HER2-amplified metastatic breast cancer (https://clinicaltrials.gov/ct2/show/NCT03379428?ter=ibrutinib&cond=Breast+Cancer&rank=1). Specifically, Ibrutinib and AVL-292 are two irreversible inhibitors that target the same critical Cys481 residue in the kinase domain [40, 41], a residue conserved also in the EGFR family members. Previous reports described an anti-proliferative or pro-apoptotic effect of Ibrutinib in EGFR-mutated NSCLC cells [34–36] that was attributed to the inhibition of mutated EGFR by Ibrutinib. Among those, Gao et al. [34] investigated BTK expression in NSCLC cell lines (among which NIH-H1975) and they did not detect p65BTK probably because they used a commercial antibody raised against the p77 isoform. Therefore, the authors did not address the inhibition of BTK by Ibrutinib. In contrast, our data indicate that the effect of Ibrutinib in NSCLC is due to p65BTK inhibition for several reasons: i) we strengthened and confirmed our results on the biological effects of p65BTK inhibition with two other inhibitors (AVL-292 and RN486), one of which has a different mechanism of action (see below); ii) all the BTK-TKIs we tested have an anti-proliferative activity independently of the EGFR mutational status in NSCLC cells (Figs. 4, 5) in contrast with the fact that Ibrutinib binds only mutant, and not wt EGFR [36]; iii) BTK-TKIs showed a synergic effect with EGFR-TKIs turning a mild anti-proliferative effect in a cytotoxic one (Fig. 6). In particular, the synergistic effect of such combination may be explained by the fact that the inhibitors act at different levels of the pathway downstream the EGFR and p65BTK acts downstream of the RAS/MAPK cascade.

Notably, given that our data show that only the p65 isoform is expressed in lung cancer cells and tissues, a p65BTK specific antibody should be used to identify potential NSCLC patients candidate for anti-BTK therapy.

Finally, our in vitro results indicate that among the BTK inhibitors RN486 is more powerful than Ibrutinib and AVL-292 in blocking lung cancer cell proliferation and sensitizing drug-resistant NSCLC cells to either EGFR-TKIs and SOC therapy. Two main reasons may explain the potency of RN486: i) RN486 is a reversible allosteric BTK inhibitor that interact with K430 [42], a residue critical for protein kinase activity [43], and does not cross react with EGFR family member, being therefore specific for BTK only; ii) Ibrutinib is metabolized and inactivated by the two isoforms of the CYP3 detoxifying enzyme 3A4 and 3A5 [44], both of them overexpressed in NSCLC [45]. This information might be relevant in the planning of clinical trials with BTK inhibitors.

In summary, we indicate that p65BTK is a putative theranostic marker in NSCLC in non-smoker patients with EGFR-wt AdC. Furthermore, our in vitro data show a synergistic effect of BTK inhibitors with targeted therapy and SOC chemotherapy in NSCLC treatment. In fact, the addition of BTK inhibitors to EGFR-targeted therapy or chemotherapy is effective in re-sensitizing NSCLC cells with an EGFR-wt and defects in the RAS/MAPK pathway, thus scarcely responsive to current treatments. Further studies are needed to better disclose mechanism of action of BTK inhibitors and criteria to accurately stratify NSCLC patients eligible for anti-BTK therapy.

## Conclusions

Our results indicate that p65BTK is a potential therapeutic target in advanced NSCLC. In the clinical setting, p65BTK inhibition might be an effective strategy for overcoming resistance of NSCLC to chemotherapy and targeted therapy in lung adenocarcinoma patients.

## Additional files

Additional file 1: **Figure S1.** BN30 antibody characterization. a Western blot analysis of lysates from HCT116p53KO cells harvested 48hs after transfection with control (Luc) or p65BTK-specific (BTK) siRNA and used to produce cells blocks. **b** IHC using BN30 on slides from cells blocks; bar: 50 μM. 40X magnification. c Western blot analysis of lysates from SW480 cells harvested 48hs after transfection with control (Luciferase) or p65BTK-specific (BTK) siRNA and from B-cell lymphoblastic leukemia cell line Nalm-6, which expresses both p65 and p77BTK. BD#611117: anti-BTK antibody from Becton Dickinson raised against the N-term of the protein and not cross-reacting with p65BTK (PDF 1492 kb)

Additional file 2: **Table S1.** Clinicopathological characteristics of NSCLC patients (*n* = 383). LGT: Lepidic growth type; AdC: adenocarcinoma; LC, large cell AdC; SCC: squamous cell carcinoma; AdC/SCC, mixed adeno-squamous carcinoma; R, rearranged. pTx or pNx, this information could not be established. (PDF 72 kb)

Additional file 3: **Figure S2.** p65BTK expression in NSCLC or AdC non-smoker patients stratified by ALK translocation (T) (PDF 247 kb)

Additional file 4: **Figure S3.** p65BTK expression in T cells. FACS-purified CD3 cell lysate was tested for p65BTK expression by BN49 antibody and for p77BTK expression using the anti-BTK (#611117, from Becton Dickinson. 100 pg of purified p77BTK (#B4312, Sigma-Aldrich) were also loaded as a positive control. (PDF 469 kb)

Additional file 5: **Table S2.** p65BTK mRNA but not p77BTK mRNA is expressed in NSCLC cell lines. mRNA expression was evaluated by RT-PCR using primers specific for each of the two isoforms [18]. (PDF 90 kb)

Additional file 6: **Figure S4.** p65BTK is overexpressed and active in NSCLC cell lines scarcely responsive to EGFR inhibition. a Immunofluorescence staining of phosphorylated p65BTK (pBTK) in untreated human p53-null NSCLC cell lines. Nuclei were counterstained with DAPI. b Western blot analysis of phosphorylated p65BTK in NSCLC cell lines. 100 pg of purified activated form p77BTK (#B4312, Sigma-Aldrich) were also loaded as a positive control. Expression levels of total p65BTK were assessed by BN49. Vinculin was used as a loading control. c Immunofluorescence staining of pBTK after 2 h treatment of SK-Lu-1 cells with BTK inhibitors (IBRU20 = Ibrutinib 20 μM; AVL10 = AVL-292 10 μM; RN10 = RN486 10 μM). d Western blot analysis of phosphorylated p65BTK in SK-Lu-1 cells after 2 h treatment with BTK inhibitors (IBRU20 = Ibrutinib 20 μM; AVL10 = AVL-292 10 μM; RN10 = RN486 10 μM. Expression levels of total p65BTK were assessed by BN49. Vinculin was used as a loading control. (PDF 3221 kb)

Additional file 7: **Figure S5.** Cell death triggered by the combination of BTK inhibitors and target therapy or SOC chemotherapy is apoptosis. a Caspase-3/7 activation after 24 hs treatment of SK-Lu-1 and NCI-H2228 cells with the indicated drugs, as assessed by luminometric assay. Error bars represent mean ± SEM. *n* = 3. b Cell viability of SK-Lu-1 and NCI-H2228 cell lines in response to combinations of BTK inhibitor RN486 and EGFR inhibitor Gefitinib or Cisplatin. (T0 = time 0; NT = untreated; RN10 = RN486 10 μM; QVD 10 = Q-VD-OPh 10 μM; GEF20 = Gefitinib 20 μM; CIS10 = Cisplatin 10 μM). X-axis crosses in correspondence of T0 values (before starting the treatment); 72 h values are then expressed as the percentage variation relative to the initial cell number. Data are presented as mean ± SEM. *n* ≥ 3 independent experiments. (PDF 301 kb)

## Abbreviations

AdC: Adenocarcinoma
ALK: Anaplastic lymphoma kinase
BTK: Bruton tyrosine kinase
EGFR: Epidermal growth factor receptor
LCC: Large Cell Carcinomas
MET: Mesenchymal epithelial transition factor
NSCLC: Non–Small Cell lung Cancer
SCC: Squamous Cell Carcinoma
SCLC: Small Cell Lung Cancer
TKI: Tyrosin Kinase Inhibitor
WT: Wild type
