# Supplementary material for: p65BTK is a novel potential actionable target in KRAS-mutated/EGFR-wild type lung adenocarcinoma
Source: J Exp Clin Cancer Res. 2019 Jun 14;38:260. doi: 10.1186/s13046-019-1199-7 (PMC6570906; doi:10.1186/s13046-019-1199-7)
Supplement: Supplementary file 1 — Figure S1. BN30 antibody characterization. a Western blot analysis of lysates from HCT116p53KO cells harvested 48hs after transfection with control (Luc) or p65BTK-specific (BTK) siRNA and used to produce cells blocks. b IHC using BN30 on slides from cells blocks; bar: 50 μM. 40X magnification. c Western blot analysis of lysates from SW480 cells harvested 48hs after transfection with control (Luciferase) or p65BTK-specific (BTK) siRNA and from B-cell lymphoblastic leukemia cell line Nalm-6, which expresses both p65 and p77BTK. BD#611117: anti-BTK antibody from Becton Dickinson raised against the N-term of the protein and not cross-reacting with p65BTK (PDF 1492 kb) [file 13046_2019_1199_MOESM1_ESM.pdf]

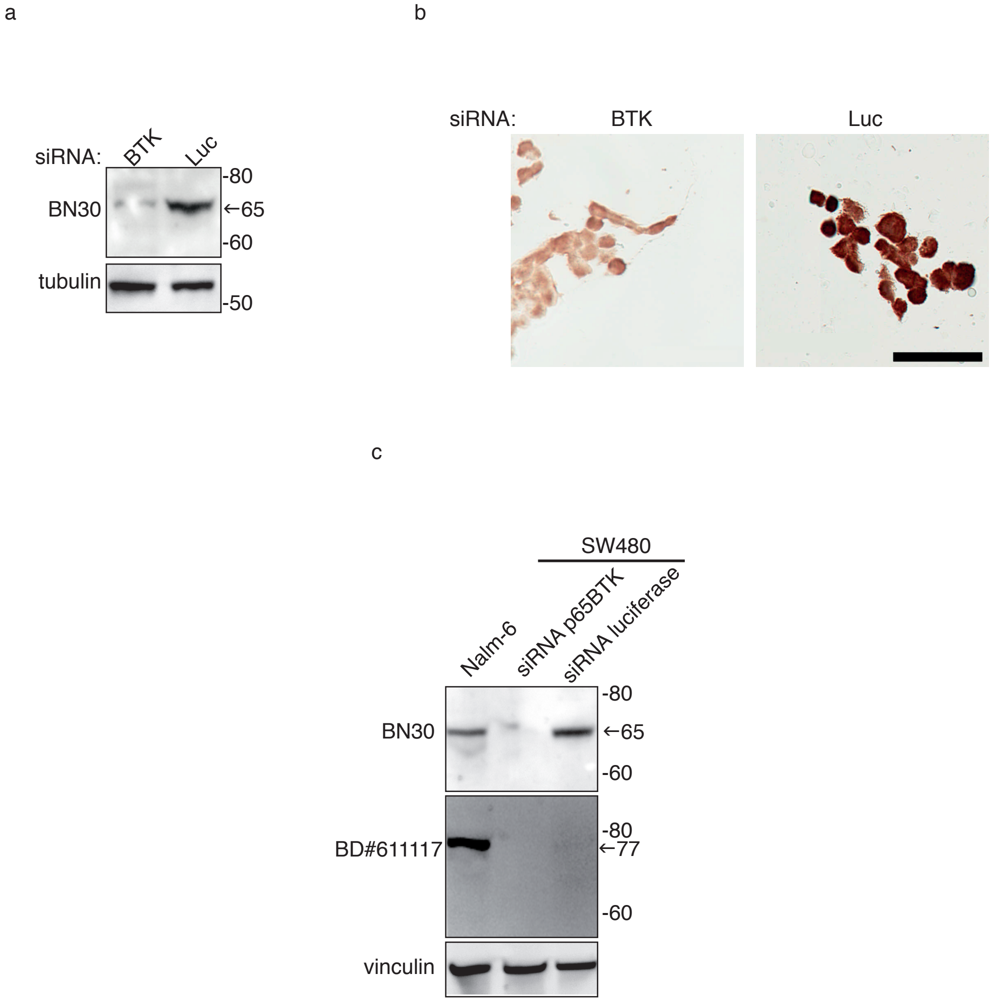

Additional file 1- Figure S1. Anti-p65BTK BN30 antibody characterization.
a Western blot analysis of lysates from SW480 cells harvested 48hs after transfection with control (Luc) or p65BTK-specific (BTK) siRNA and used to produce cells blocks.**b** IHC using BN30 on slides from cells blocks; bar: 50μM. **c** Western blot analysis of lysates from SW480 cells harvested 48hs after transfection with control (Luciferase) or p65BTK-specific (BTK) siRNA and from B-cell lymphoblastic leukemia cell line Nalm-6, whihc expresses both p65 and p77BTK. BD#611117: anti-BTK antibody from Becton Dickinson raised against the N-term of the protein and not cross-reacting with p65BTK.
