# Supplementary material for: p65BTK is a novel potential actionable target in KRAS-mutated/EGFR-wild type lung adenocarcinoma
Source: J Exp Clin Cancer Res. 2019 Jun 14;38:260. doi: 10.1186/s13046-019-1199-7 (PMC6570906; doi:10.1186/s13046-019-1199-7)
Supplement: Supplementary file 2 — Table S1. Clinicopathological characteristics of NSCLC patients (n = 383). LGT: Lepidic growth type; AdC: adenocarcinoma; LC, large cell AdC; SCC: squamous cell carcinoma; AdC/SCC, mixed adeno-squamous carcinoma; R, rearranged. pTx or pNx, this information could not be established. (PDF 72 kb) [file 13046_2019_1199_MOESM2_ESM.pdf]

	Histotype	n	Smoke (yes)	pT				pN				Grade				ALK	EGFR		OS status Censored
				1	2	3/4	x	0	1	2	x	1	2	3	na	R	L858R	Δex19	
AdC	LGT	7	6	5	1	1	-	7	-	-	-	7	-	-	-	-	-	-	7
	AdC	265	182	114	111	31	9	173	51	41	-	14	140	101	10	8	3	5	158
	LC	22	19	3	11	5	3	9	7	6	-	-	-	19	3	-	-	-	10
SCC	SCC	84	69	24	44	12	4	54	27	2	1	1	44	37	2	3	1	-	55
	AdC/SCC	5	1	2	1	2	-	2	1	2	-	-	3	2	-	-	-	-	2

Additional file 2: Table S1. Clinico-pathological characteristics of NSCLC patients (n=383). LGT: lepidic growth type; AdC: adenocarcinoma; LC, large cell AdC; SCC: squamous cell carcinoma; AdC/SCC, mixed adeno-squamous carcinoma; R, rearranged. pTx or pNx, this information could not be established.
