# Supplementary material for: p65BTK is a novel potential actionable target in KRAS-mutated/EGFR-wild type lung adenocarcinoma
Source: J Exp Clin Cancer Res. 2019 Jun 14;38:260. doi: 10.1186/s13046-019-1199-7 (PMC6570906; doi:10.1186/s13046-019-1199-7)
Supplement: Supplementary file 3 — Figure S2. p65BTK expression in NSCLC or AdC non-smoker patients stratified by ALK translocation (T) (PDF 247 kb) [file 13046_2019_1199_MOESM3_ESM.pdf]

Additional file 3 - Figure S2

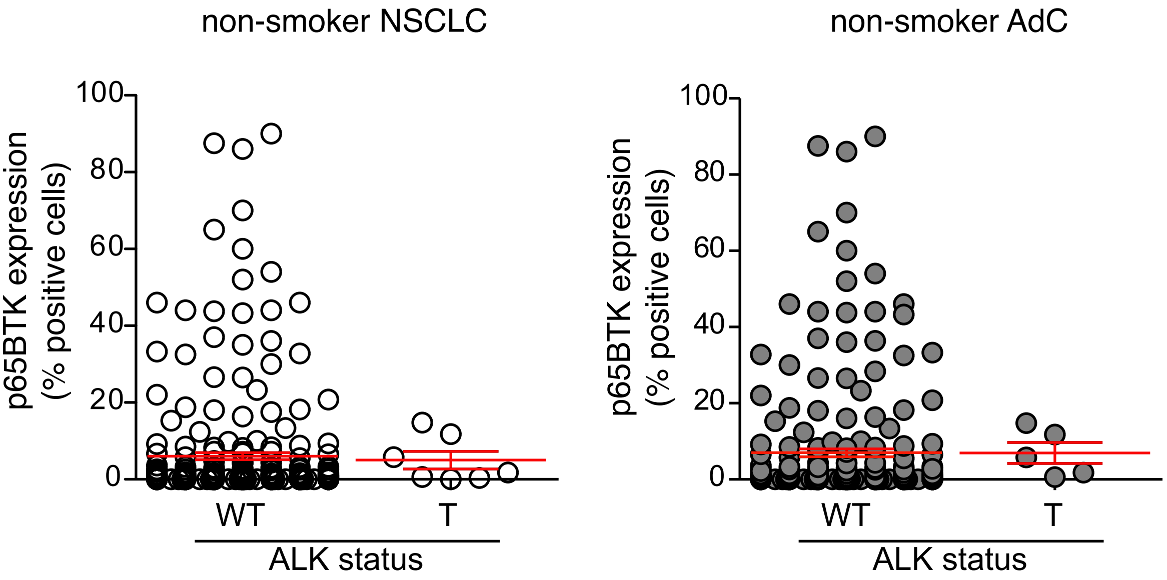

Additional file 3 - Figure S2. p65BTK expression in NSCLC or AdC non-smoker patients stratified by ALK translocation (T).
