# Supplementary material for: p65BTK is a novel potential actionable target in KRAS-mutated/EGFR-wild type lung adenocarcinoma
Source: J Exp Clin Cancer Res. 2019 Jun 14;38:260. doi: 10.1186/s13046-019-1199-7 (PMC6570906; doi:10.1186/s13046-019-1199-7)
Supplement: Supplementary file 4 — Figure S3. p65BTK expression in T cells. FACS-purified CD3 cell lysate was tested for p65BTK expression by BN49 antibody and for p77BTK expression using the anti-BTK (#611117, from Becton Dickinson. 100 pg of purified p77BTK (#B4312, Sigma-Aldrich) were also loaded as a positive control. (PDF 469 kb) [file 13046_2019_1199_MOESM4_ESM.pdf]

Additional file 4 - Figure S3

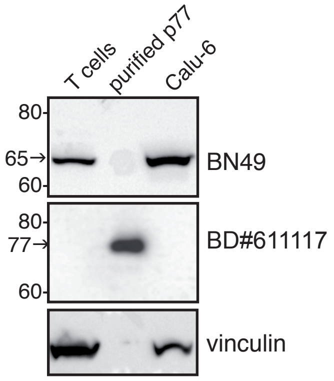

Additional file 4 - Figure S3. p65BTK expression in T cells. FACS-purified T cell lysate was tested for p65BTK expression by BN49 antibody and for p77BTK expression using the anti-BTK (#611117) from Becton Dickinson. 100pg of purified p77BTK (#B4312, Sigma- Aldrich) were also loaded as a positive control.
