# Supplementary material for: p65BTK is a novel potential actionable target in KRAS-mutated/EGFR-wild type lung adenocarcinoma
Source: J Exp Clin Cancer Res. 2019 Jun 14;38:260. doi: 10.1186/s13046-019-1199-7 (PMC6570906; doi:10.1186/s13046-019-1199-7)
Supplement: Supplementary file 5 — Table S2. p65BTK mRNA but not p77BTK mRNA is expressed in NSCLC cell lines. mRNA expression was evaluated by RT-PCR using primers specific for each of the two isoforms [18]. (PDF 90 kb) [file 13046_2019_1199_MOESM5_ESM.pdf]

	CYCLE THRESHOLD	
	**p65**	**p77**
**Calu-6**	33 ± 0.5	undetermined
**SK-Lu-1**	34 ± 0.1	undetermined
**NCI-H1975**	34 ± 0.2	undetermined
**NCI-H2228**	34 ± 0.3	undetermined

Additional file 5: Table S2. p65BTK mRNA but not p77BTK mRNA is expressed in NSCLC cell lines. mRNA expression was evaluated by RT-PCR using primers specific for each of the two isoforms (18).
