# Supplementary material for: p65BTK is a novel potential actionable target in KRAS-mutated/EGFR-wild type lung adenocarcinoma
Source: J Exp Clin Cancer Res. 2019 Jun 14;38:260. doi: 10.1186/s13046-019-1199-7 (PMC6570906; doi:10.1186/s13046-019-1199-7)
Supplement: Supplementary file 6 — Figure S4. p65BTK is overexpressed and active in NSCLC cell lines scarcely responsive to EGFR inhibition. a Immunofluorescence staining of phosphorylated p65BTK (pBTK) in untreated human p53-null NSCLC cell lines. Nuclei were counterstained with DAPI. b Western blot analysis of phosphorylated p65BTK in NSCLC cell lines. 100 pg of purified activated form p77BTK (#B4312, Sigma-Aldrich) were also loaded as a positive control. Expression levels of total p65BTK were assessed by BN49. Vinculin was used as a loading control. c Immunofluorescence staining of pBTK after 2 h treatment of SK-Lu-1 cells with BTK inhibitors (IBRU20 = Ibrutinib 20 μM; AVL10 = AVL-292 10 μM; RN10 = RN486 10 μM). d Western blot analysis of phosphorylated p65BTK in SK-Lu-1 cells after 2 h treatment with BTK inhibitors (IBRU20 = Ibrutinib 20 μM; AVL10 = AVL-292 10 μM; RN10 = RN486 10 μM. Expression levels of total p65BTK were assessed by BN49. Vinculin was used as a loading control. (PDF 3221 kb) [file 13046_2019_1199_MOESM6_ESM.pdf]

Additional file 6 - Figure S4

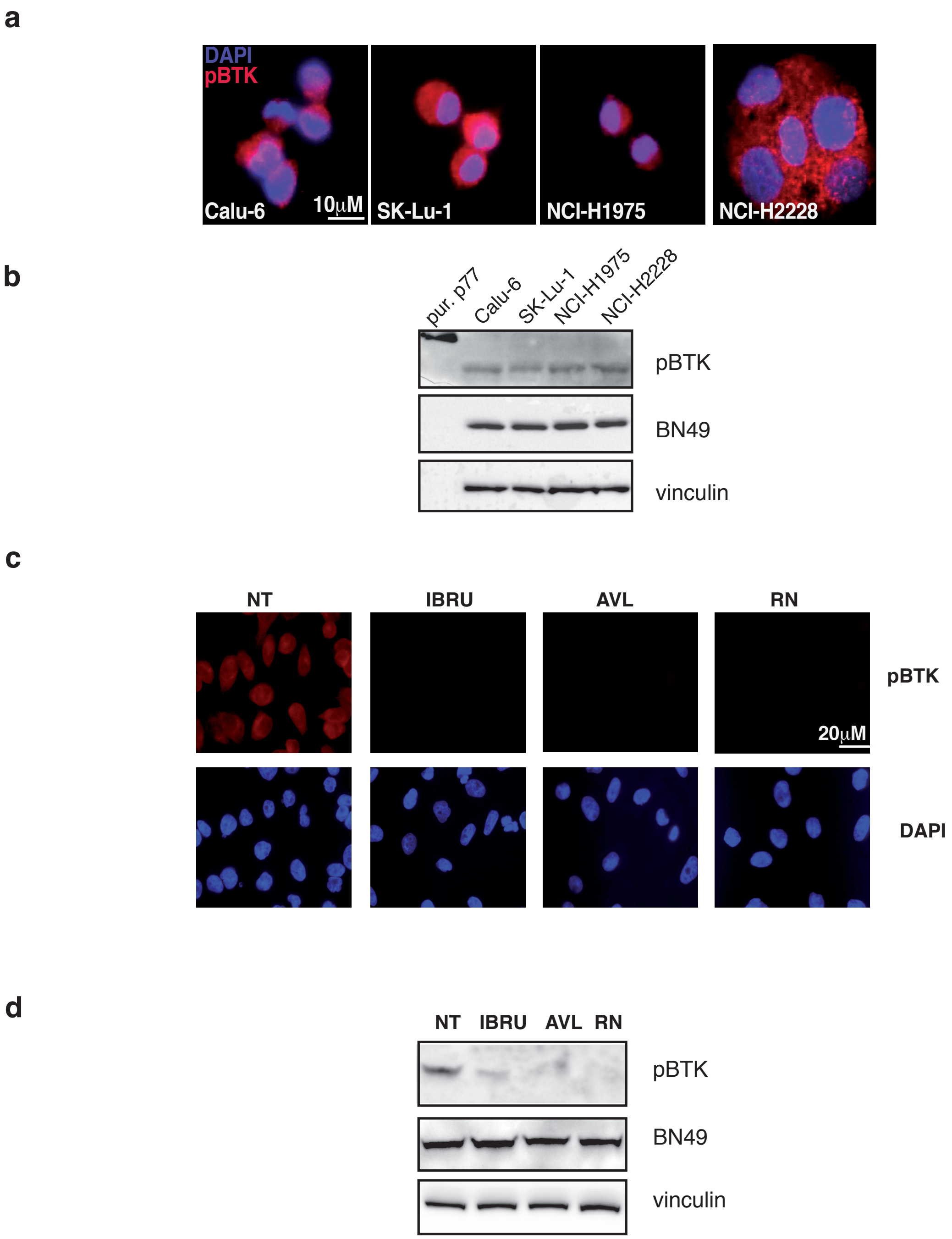

Additional file 6- Figure S4. p65BTK is overexpressed and active in NSCLC cell lines scarcely responsive to EGFR inhibition. a Immunofluorescence staining of phosphorylated p65BTK (pBTK) in untreated human p53-null NSCLC cell lines. Nuclei were counterstained with DAPI. **b** Western blot analysis of phosphorylated p65BTK in NSCLC cell lines. 100pg of purified and activated p77BTK (#B4312, Sigma-Aldrich) were also loaded as a positive control. Levels of total p65BTK were assessed by BN49. Vinculin was used as a loading control. **c** Immunofluorescence staining of pBTK after 2 hours treatment of SK-Lu-1 cells with BTK inhibitors (IBRU20 = Ibrutinib 20 μM; AVL10 = AVL-292 10 μM; RN10 = RN486 10 μM). **d** Western blot analysis of phosphorylated p65BTK in SK-Lu-1 cells after 2 hours treatment with BTK inhibitors (IBRU20 = Ibrutinib 20 μM; AVL10 = AVL-292 10 μM; RN10 = RN486 10 μM. Levels of total p65BTK were assessed by BN49. Vinculin was used as a loading control
