# Supplementary material for: p65BTK is a novel potential actionable target in KRAS-mutated/EGFR-wild type lung adenocarcinoma
Source: J Exp Clin Cancer Res. 2019 Jun 14;38:260. doi: 10.1186/s13046-019-1199-7 (PMC6570906; doi:10.1186/s13046-019-1199-7)
Supplement: Supplementary file 7 — Figure S5. Cell death triggered by the combination of BTK inhibitors and target therapy or SOC chemotherapy is apoptosis. a Caspase-3/7 activation after 24 hs treatment of SK-Lu-1 and NCI-H2228 cells with the indicated drugs, as assessed by luminometric assay. Error bars represent mean ± SEM. n = 3. b Cell viability of SK-Lu-1 and NCI-H2228 cell lines in response to combinations of BTK inhibitor RN486 and EGFR inhibitor Gefitinib or Cisplatin. (T0 = time 0; NT = untreated; RN10 = RN486 10 μM; QVD 10 = Q-VD-OPh 10 μM; GEF20 = Gefitinib 20 μM; CIS10 = Cisplatin 10 μM). X-axis crosses in correspondence of T0 values (before starting the treatment); 72 h values are then expressed as the percentage variation relative to the initial cell number. Data are presented as mean ± SEM. n ≥ 3 independent experiments. (PDF 301 kb) [file 13046_2019_1199_MOESM7_ESM.pdf]

Additional file 7 - Figure S5

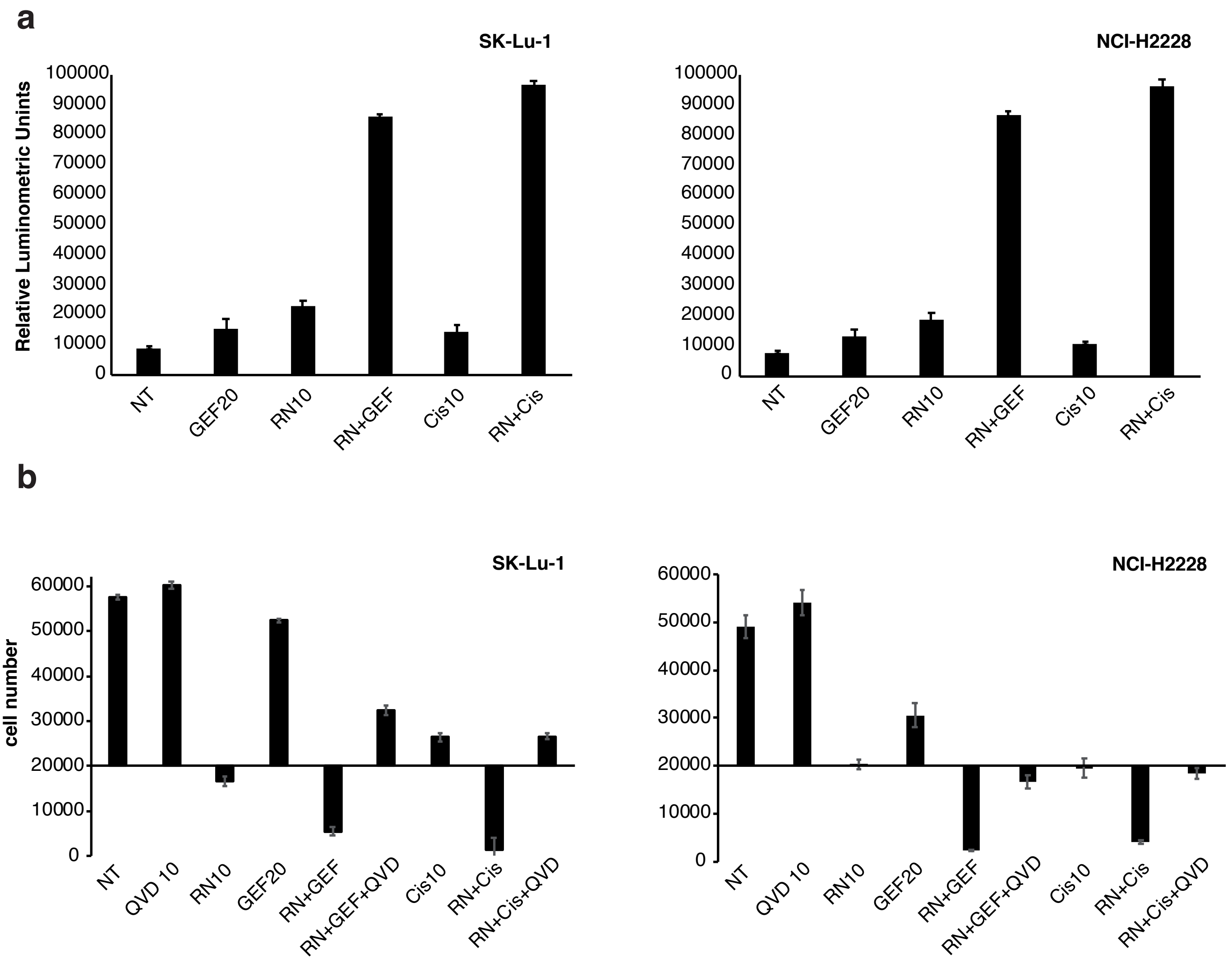

Additional file 7 - Figure S5. Cell death triggered by the combination of BTK inhibitors and target therapy or SOC chemotherapy is apoptosis. a Caspase-3/7 activation after 24 hs treatment of SK-Lu-1 and NCI-H2228 cells with the indicated drugs as assessed by a luminometric assay. Error bars represent mean ± SEM. n = 3. **b** Cell viability of SK-Lu-1 and NCI-H2228 cell lines in response to combinations of BTK inhibitor RN486 and EGFR inhibitor Gefitinib or Cisplatin. (T0 = time 0; NT = untreated; RN10= RN486 10 μM; QVD 10 = Q-VD-OPh 10 μM; GEF20 = Gefitinib 20 μM; CIS10 = Cisplatin 10 μM). X-axis crosses in correspondence of T0 values (before starting the treatment); 72 hours values are then expressed as the percentage variation relative to the initial cell number. Data are presented as mean ± SEM. n ≥ 3 independent experiments.
